# Supplementary material for: Identification and Functional Analysis of bZIP Genes in Cotton Response to Drought Stress
Source: Int J Mol Sci. 2022 Nov 28;23(23):14894. doi: 10.3390/ijms232314894 (PMC9736030; doi:10.3390/ijms232314894)
Supplement: Supplementary file 1 [file ijms-23-14894-s001.zip › ijms-2016824-SM figures.pdf]

International Journal of
Molecular Sciences

MDPI

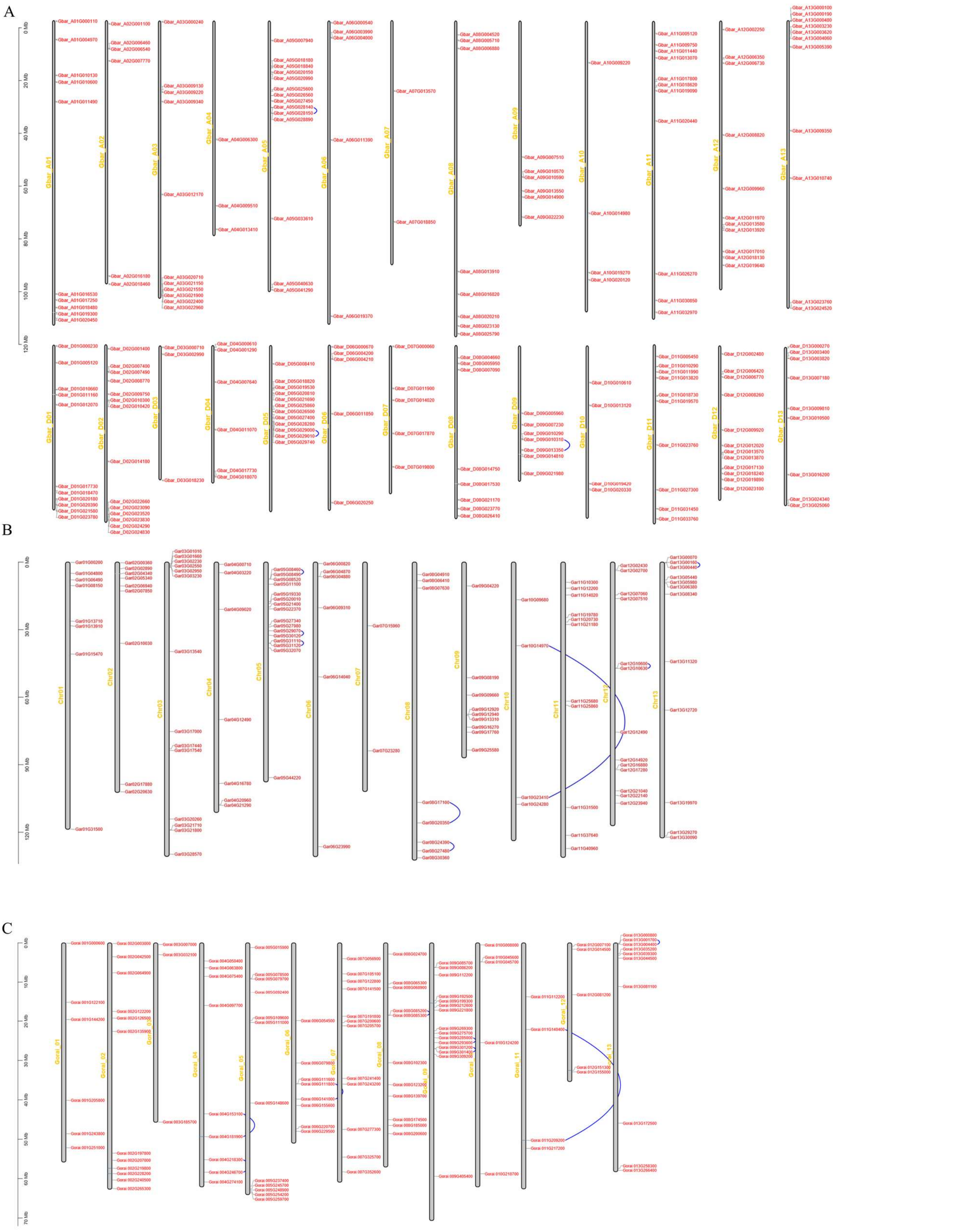

Figure S1. Genomic distribution of bZIP genes on the chromosomes of G. barbardense, G.arboreum and G. raimondii.

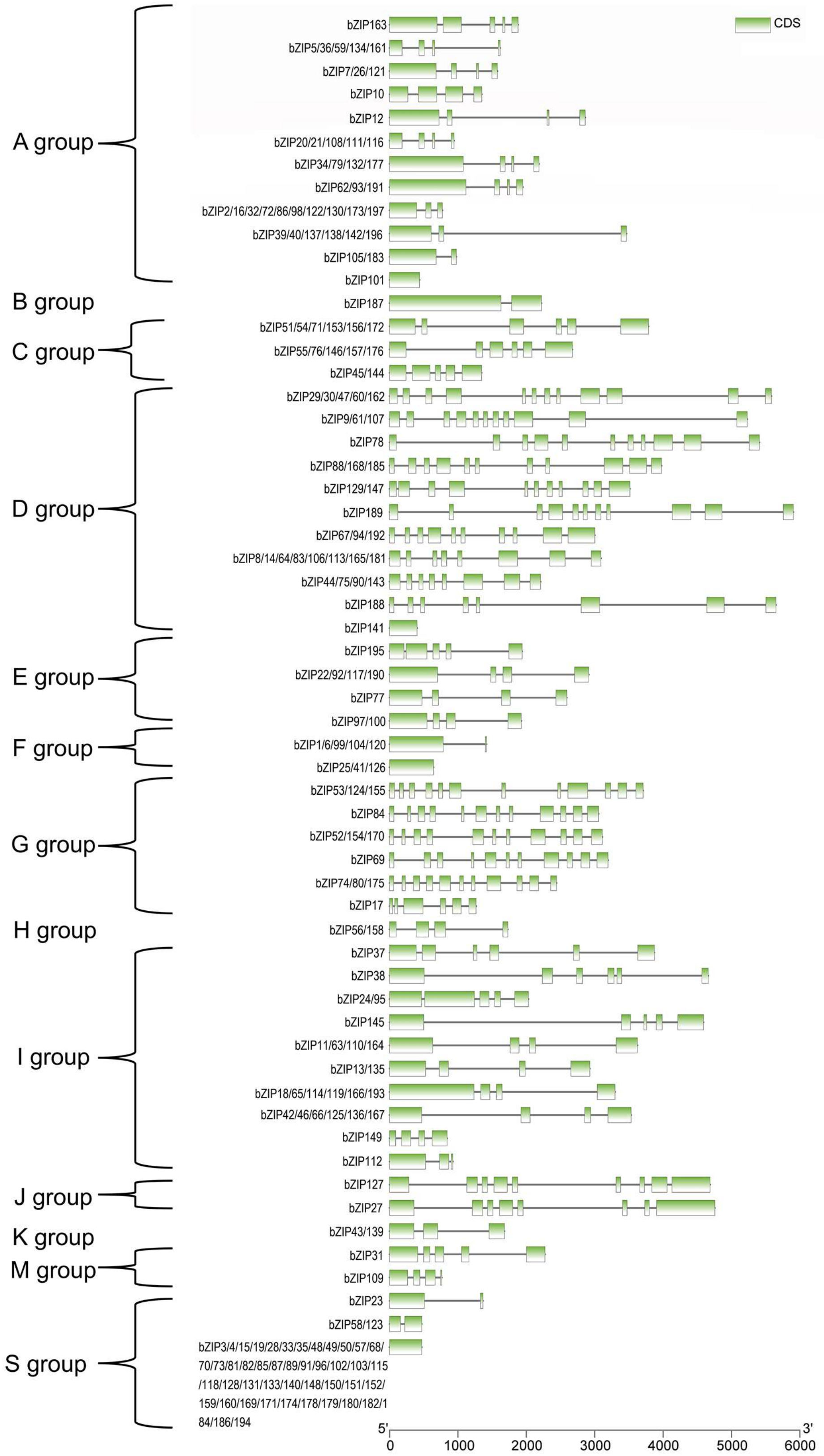

Figure S2. Exon/intron organization of GhbZIP genes depicted for each group.

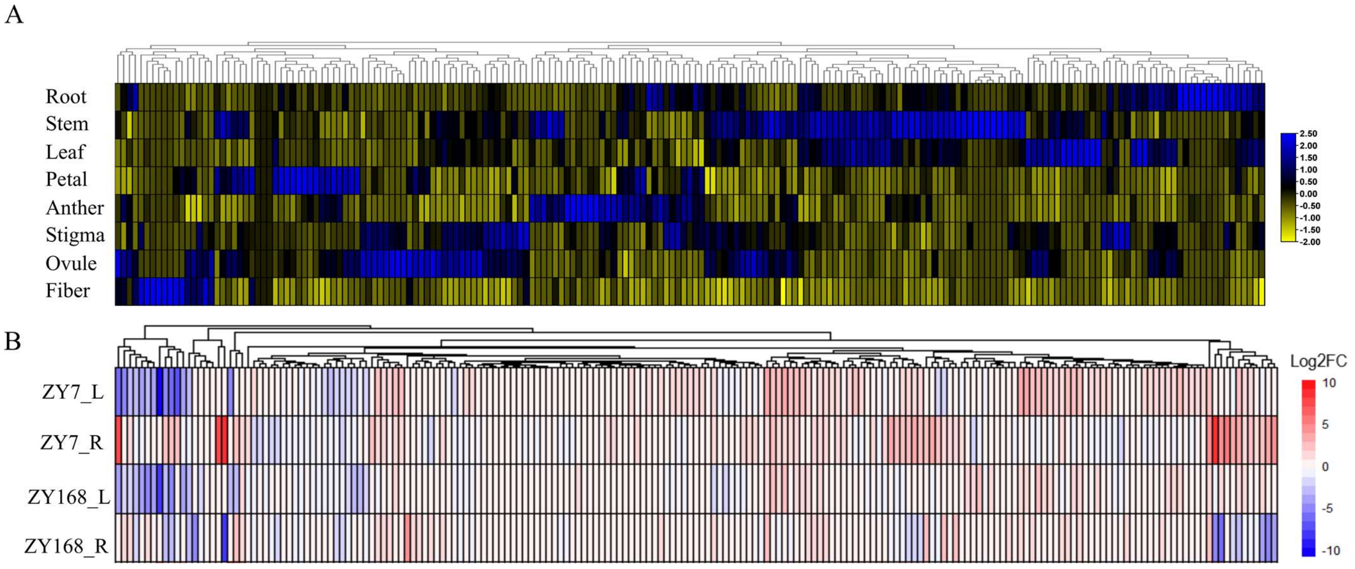

Figure S3. The expression patterns of GhbZIP genes in different tissues/organs and under drought treatment.
